# The effect of cognitive reappraisal and early-life maternal care on neuroendocrine stress responses

**DOI:** 10.1038/s41598-024-57106-x

**Published:** 2024-03-21

**Authors:** Ulrike U. Bentele, Elea S. C. Klink, Annika B. E. Benz, Maria Meier, Raphaela J. Gaertner, Bernadette F. Denk, Stephanie J. Dimitroff, Eva Unternaehrer, Jens C. Pruessner

**Affiliations:** 1 Department of Psychology, Division of Neuropsychology, University of Konstanz, Universitaetsstrasse 10, 78464 Constance, Germany; 2 Centre for the Advanced Study of Collective Behaviour, University of Konstanz, Constance, Germany; 3 Child- and Adolescent Research Department, University Psychiatric Clinics Basel (UPK), University of Basel, Basel, Switzerland; 4 Department of Psychology, University of Montana, Montana, 59812 USA

**Keywords:** Psychology, Biomarkers, Endocrinology

## Abstract

Early-life adversity (ELA) is related to profound dysregulation of the hypothalamic–pituitary–adrenal (HPA) axis, reflected in both, blunted or exaggerated cortisol stress responses in adulthood. Emotion regulation strategies such as cognitive reappraisal might contribute to this inconsistent finding. Here, we investigate an interaction of early-life maternal care (MC), where low MC represents a form of ELA, and instructed emotion regulation on cortisol responses to acute stress. Ninety-three healthy young women were assigned to a low (*n* = 33) or high (*n* = 60) MC group, based on self-reported early-life MC. In the laboratory, participants received regulation instructions, asking to cognitively reappraise (reappraisal group, *n* = 45) or to focus on senses (control group, *n* = 48) during subsequent stress exposure, induced by the Trier Social Stress Test. Salivary cortisol and subjective stress levels were measured repeatedly throughout the experiment. Multilevel model analyses confirmed a MC by emotion regulation interaction effect on cortisol trajectories, while controlling for hormonal status. Individuals with low MC in the control compared with the reappraisal group showed increased cortisol responses; individuals with high MC did not differ. These results highlight the significance of emotion regulation for HPA axis stress regulation following ELA exposure. They provide methodological and health implications, indicating emotion regulation as a promising target of treatment interventions for individuals with a history of ELA.

## Introduction

Exposure to adverse early-life experiences is a major risk factor for mental and physical disease later in life^1,2^. One key mechanism considered to underlie this association is a dysregulation of the main neuroendocrine stress system, the hypothalamic–pituitary–adrenal (HPA) axis^3–5^. Precise regulation of the HPA axis and associated release of the glucocorticoid cortisol, that mediates vital metabolic and cardiovascular processes, is essential to adaptively respond and recover from acute stress^6,7^. Dysregulation of the HPA axis, reflected in hyper- or hypoactive stress responsivity, is thereby linked to poor health or disease^8,9^. Such patterns of HPA axis dysregulation can result from chronic stress exposure^10,11^, such as exposure to early-life adversity (ELA).

### Early-life adversity and HPA axis reactivity

ELA refers to a variety of adverse and stressful experiences that happen within the social environment during childhood or adolescence^12,13^. Besides abuse and neglect^14^, the relationship between child and caregiver, such as quality of care, has been identified as key dimension of ELA^15,16^. Care—specifically provided by the mother—is considered the most critical developmental influence of early-life environment^17,18^. While high maternal care (MC) refers to a mother’s caring, responsive and sensitive behaviors towards her child, low MC in turn describes insensitive and unpredictable behavior patterns^19,20^.

Exposure to ELA, such as low MC^19,21^, during sensitive periods of brain development has been assumed to contribute to long-lasting alterations of HPA axis reactivity^10,22^. Extensive empirical research has confirmed the link between ELA and dysregulated HPA axis stress responses^23^. The direction of this dysregulation is however more inconsistent and possible factors determining the direction of the dysregulation are under continuous investigation. Some studies reported reduced cortisol responses to psychosocial stress in adults exposed to ELA, such as neglect or abuse (e.g.,^24,25^); others found increased^26,27^ or no association with cortisol stress responses^28^. With respect to maternal parenting, there is evidence that low levels of MC might mainly result in patterns of blunted cortisol reactivity (^18,29^; but see^30^). For example, individuals reporting a low extent of MC showed reduced cortisol reactivity to psychosocial stress compared to those reporting a moderate extent of MC^29^.

In sum, exposure to ELA has persistently been related to HPA axis dysregulation in adulthood, with a low extent of MC mainly resulting in reduced cortisol stress responses. One factor that might contribute to explain the inconsistency is emotion regulation capacity^31,32^, specifically the use of cognitive reappraisal.

### Regulation strategies and HPA axis reactivity

Emotion regulation (ER) refers to processes by which individuals attempt to influence the nature, intensity, duration or expression of emotions^33,34^. Emotions are thereby considered to unfold over time, starting with exposure to an emotional situation and terminating in the final emotional response, characterized at the subjective, behavioral and physiological level^35,36^. Regulation can occur at multiple stages of this emotion generation process, thereby affecting the final psychophysiological response. One central point of regulation is appraisal of the personal meaning that is attributed to the situation^37^. For instance, if a situation is appraised as irrelevant to the personal well-being no subsequent reaction will occur^38^. Cognitive *re*appraisal, in contrast, describes the re-interpretation of the situation to alter its emotional impact^39^. Here, individuals might try to focus on positive aspects of an emotional aversive situation or adopt an objective third-person perspective^40,41^. Cognitive reappraisal is thereby considered an adaptive ER strategy^42,43^, that shows negative relations with psychopathology and positive associations with mood and well-being^44,45^.

Empirical work has consistently revealed cognitive reappraisal to reduce subjective responses to emotion or stress eliciting situations, whereas effects on neuroendocrine or physiological responses are small at best, as reviewed in^41,46, 47^. While the number of studies on the biological (particularly neuroendocrine) effects of reappraisal is rather small, results are partly heterogeneous. Here, different moderators may play a role (e.g., type of stressor, outcome measure)^46^. Studies that have specifically focused on HPA axis stress responses to acute psychosocial stress imply that cognitive reappraisal can increase cortisol responses. For instance, individuals who reported higher habitual use of reappraisal (trait reappraisal) exhibited stronger cortisol reactivity to psychosocial stress^48,49^. This was further confirmed by three experimental studies^50–52^. Individuals who were instructed to use reappraisal during a psychosocial or physical stress task, e.g., by adopting an unemotional third-person perspective compared to individuals who received no instruction, exhibited increased cortisol responses^50^. Interestingly, this amplifying effect of instructed reappraisal was found predominantly in individuals with lower trait reappraisal – which the authors discuss as requiring higher cognitive effort and associated increased physiological activation when following a reappraisal instruction in individuals who do not use reappraisal regularly. In sum, past work provides some evidence that instructed cognitive reappraisal might increase HPA axis responses to acute stress.

### ELA, regulation strategies and HPA axis reactivity

The identification of factors that contribute to the heterogeneous results concerning the effect of ELA is of major scientific interest as it might reveal novel targets for intervention^11,53^. Here, we investigate adaptive ER as one such factor: Cognitive reappraisal has been shown to have the potential to increase cortisol responses to psychosocial stress—particularly when not used regularly^51,52^. Early-life stress is related to less use of adaptive ER and reappraisal^44^. Thus, being instructed to use cognitive reappraisal might prove specifically beneficial for high ELA individuals, to normalize dysregulated, mainly blunted, cortisol reactivity to acute stress.

Support for the proposed interaction between ELA and ER stems from prior empirical and theoretical work. First, there is evidence that high ER capacity might allow individuals exposed to ELA to show robust cortisol responses. For example, women with a history of ELA who reported greater ER difficulties compared to those with less ER difficulties displayed blunted cortisol responses to psychosocial stress; women with low ELA did not differ depending on ER capacity^54^. However, ER capacity does not always affect cortisol stress responses of individuals with high ELA^55^. Second, the effect of instructed ER is assumed to depend on personality characteristics (e.g., emotional intelligence)^56^. Personal experiences such as ELA have been proposed another impact factor, to be tested in future studies^47^. We expanded on these findings by testing the effects of ELA and instructed reappraisal on cortisol stress responses, while accounting for trait reappraisal. Finally, an interaction of ELA and ER could be based on similar cerebral mechanisms, such as an involvement of the prefrontal cortex (PFC). Since the use of cognitive reappraisal requires activation of prefrontal networks^57,58^, alterations in these structures as a consequence of ELA^59,60^ might in turn affect downstream HPA axis reactivity in individuals with high ELA.

The aim of the current study was thus to test the effect of experimentally induced reappraisal and naturally occurring variations in ELA on cortisol responses to acute psychosocial stress in healthy young women. For this, we first screened participants for exposure to ELA, operationalized by the extent of self-reported early-life *MC*, and categorized them to either a low or high MC group. During the laboratory sessions, participants were then exposed to psychosocial stress, induced by the Tier Social Stress Test (TSST)^61^. Prior to stress exposure, they received instructions regarding the use of emotion *regulation* strategies, asking them either to reappraise the stress task (e.g., by thinking of its positive aspects) (reappraisal group (RG)) or to focus on their senses (control group (CG)). Salivary cortisol and subjective stress levels were assessed seven times in predefined intervals; state affect was assessed three times throughout the experiment. We hypothesized that MC and emotion regulation would interact in affecting cortisol stress responses. Specifically, we expected that individuals with low MC in the reappraisal compared to the control group would show increased cortisol stress responses similarly to individuals with high MC. Moreover, we explored the effects of MC and regulation on subjective-emotional responses, comprising subjective stress and affect ratings.

## Results

### Sample characteristics

The final sample consisted of *N* = 93 women (*M*_*age*_ = 21.16, *SD*_*age*_ = 2.78, range = 18–28) with groups comprising *n* = 17 (low MC/CG), *n* = 16 (low MC/RG), *n* = 31 (high MC/CG) and *n* = 29 (high MC/RG) participants. One-way ANOVAs revealed significant group differences with regard to age (*F*(3,89) = 3.50, *p* = 0.019, η_p_^2^ = 0.105), depressive symptoms (BDI-II) (*F*(3,89) = 4.73, *p* = 0.004, η_p_^2^ = 0.137), and self-esteem (*F*(3,89) = 3.58, *p* = 0.017, η_p_^2^ = 0.108). Post-hoc t-tests indicated (1) higher age in the low MC & reappraise group compared to both the high MC groups (ps <  = 0.032), (2) higher depressiveness in the low MC & control group compared to both the high MC groups (ps <  = 0.029), and (3) lower self-esteem in the low MC & reappraise compared to the high MC & reappraise group (p = 0.031). For descriptive and inferential statistics see Table 1.

**Table 1:** Participant characteristics and comparisons of the four groups (N = 93).

MC	Regulation condition					
	CG		RG			
	High	Low	High	Low	p^b^	
*n*	31	17	29	16		
PBI maternal care	32.94(2.46)	22.71(4.27)	32.62(2.69)	18.69(5.56)	** < .001**	
age (yr)	20.77(2.50)	20.82(2.48)	20.69(2.35)	23.13(3.59)	.**019**	
BMI (kg/m^2^)	21.01(1.75)	21.64(2.97)	20.77(2.51)	22.36(2.72)	.163	
hormonal status^a^ (FP/LP/OC/UP)	11/7/7/6	4/7/5/1	3/9/11/6	3/4/5/4	.448	
BDI-II	5.42(4.84)	9.71(5.31)	5.28(4.60)	8.94(5.20)	**.004**	
RSES	23.26(4.96)	20.82(5.98)	23.86(4.22)	19.31(5.79)	**.017**	
ERQ reappraisal suppression	5.18(0.65) 3.17(1.10)	4.99(1.06) 3.09(1.23)	5.11(0.93) 2.85(0.90)	4.96(0.87) 3.78(1.44)	.818 .080	

Mean values (± standard deviations) or absolute frequencies of participant characteristics. Significant differences are depicted in bold. Bonferroni-corrected post-hoc t-tests are reported together with sample characteristics in the text. CG = control group, RG = reappraisal group, MC = maternal care, BMI = body mass index, FP = follicular phase, LP = luteal phase, UP = unclear phase, OC = use of oral contraceptives, PBI = Parental Bonding Instrument, BDI-II = Beck Depression Inventory, RSES = Rosenberg Self-Esteem Scale, ERQ = Emotion Regulation Questionnaire.

^a^At day of testing.

^b^p-values result from one-way ANOVA (for age, BMI, PBI, BDI, ERQ, RSES) and Fisher’s exact test (hormonal status).

### Manipulation checks

#### Regulation instruction

Descriptive evaluation of two manipulation check items suggested accurate understanding of regulation instructions. This was indicated by high disagreement with one item providing an incorrect description of the regulation instruction (*M* = 1.24, *SD* = 0.67), and agreement with another item providing a correct description (*M* = 4.78, *SD* = 0.76). Both items were answered on a Likert scale ranging from 1 (*not at all*) to 5 (*very much*). Kruskal–Wallis tests did not show differences between groups with regard to the incorrect (H(3) = 3.69,* p* = 0.297), but with regard to the correct item (H(3) = 31.09, *p* < 0.001). Post-hoc tests indicated lower agreement reflecting worse understanding in the low MC & reappraise group compared to the other three groups (*p*s <  = 0.001). In sum, based on the qualitative and quantitative approach to assess understanding (see methods), results indicated an overall high understanding of the instructions.

#### Stress induction

**Figure 1:**
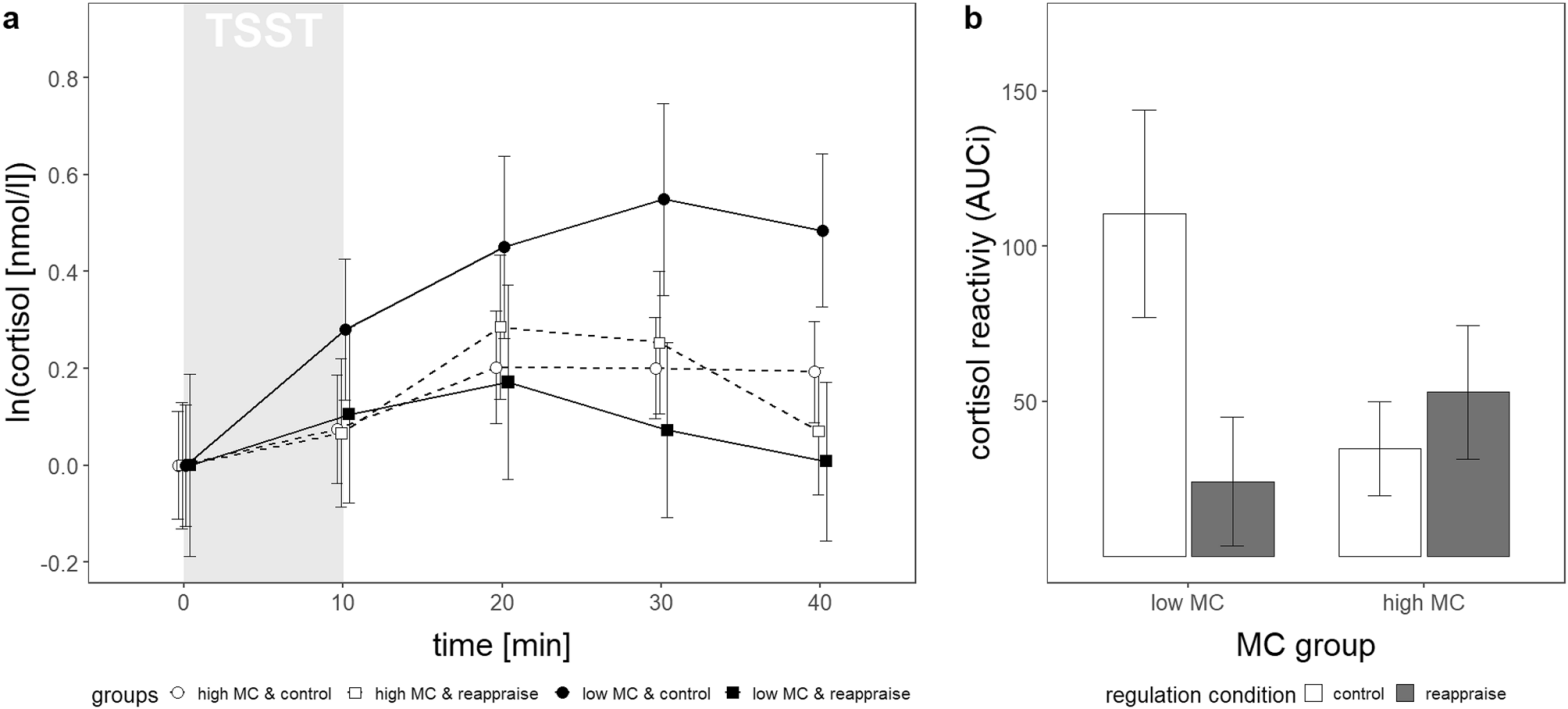
Cortisol trajectories (baseline adjusted) (**a**) and cortisol reactivity (**b**) in the four experimental groups. Values represent mean ± *SE*. The AUCi indicates overall cortisol reactivity between t = 0 and t = 40. AUCi = area under the curve with respect to increase, MC = maternal care, TSST = Trier Social Stress Test.

Cortisol trajectories were best described by a basic growth curve model (GCM) with random intercepts, random slopes, and a fixed linear and quadratic trend of time (see Supplementary Tables S2, S3). The quadratic trend of time (*b* = − 1.41, *SE* = 0.37, *t*(362) = − 3.80, *p* < 0.001) indicated an increase of cortisol levels after TSST cessation (between t =  + 10 and t =  + 20, *p* = 0.005, post-hoc t-tests) and subsequent decrease, which is depicted in Fig. 1a.

**Figure 2:**
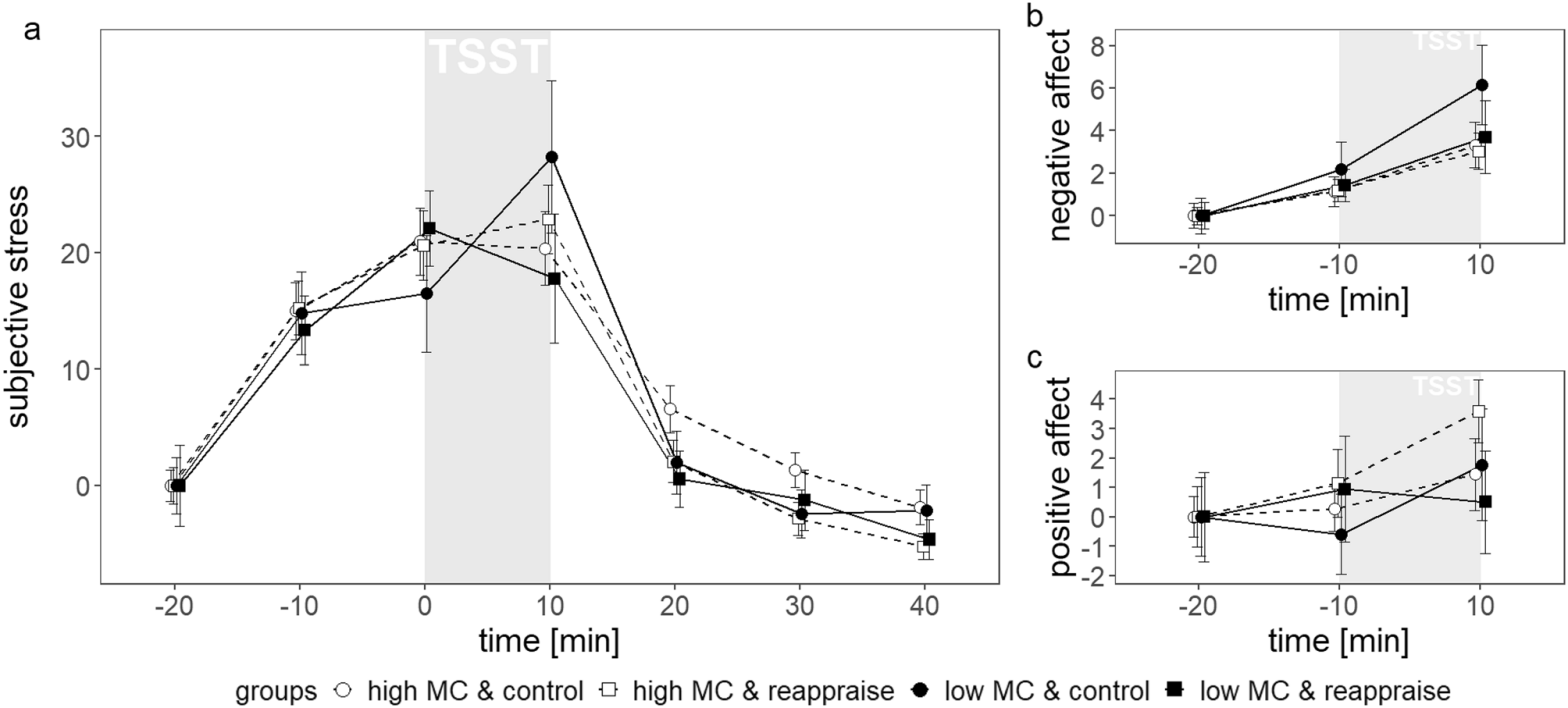
Trajectories of subjective stress (**a**), negative affect (**b**) and positive affect levels (**c**) in the four experimental groups. Values (baseline adjusted) represent mean ± *SE*. MC = maternal care, TSST = Trier Social Stress Test.

Subjective stress trajectories were best explained by a basic model with random intercepts, random slopes, and a fixed linear, quadratic and cubic trend of time (see Supplementary Tables S4, S5). Following the cubic trend of time (b = 115.45, SE = 12.08, t(555) = 9.56, p < 0.001), stress levels increased prior to TSST onset (between t = − 20 and t = 0; ps <  = 0.012) and decreased in the first part of recovery (between t =  + 10 and t =  + 30; ps < 0.001; post-hoc t-tests), as illustrated in Fig. 2a.

Thus, across groups the TSST effectively induced subjective stress and cortisol stress responses.

### Effect of MC and regulation on cortisol responses

#### Cortisol trajectories using repeated measures of cortisol over time

To examine the hypothesized three-way interaction, we added MC, regulation and higher-order interactions to the basic model, followed by separate inclusion of each covariate and covariate by time interaction. Inclusion of the MC by regulation by time interaction did not increase model fit, but further inclusion of the hormonal status (with respect to menstrual cycle) by time interaction did (see Supplementary Tables S2, S3).

Evaluation of this final interaction model revealed a significant linear trend of *time* (*b* = 3.36, *SE* = 1.38, *t*(350) = 2.44, *p* = 0.015), a time by regulation (*b* = − 3.90, *SE* = 1.61, *t*(350) = − 2.42, *p* = 0.016), time by regulation by MC (*b* = 4.09, *SE* = 1.99,* t*(350) = 2.06, *p* = 0.040), and a time by hormonal status interaction (*b* = 2.68, *SE* = 1.33, *t*(350) = 2.02, *p* = 0.044). There were no main effects of MC or regulation (*p*s >  = 0.169). To follow up the three-way interaction, the effect of regulation on cortisol trajectories was evaluated separately within the low MC and high MC group using two corresponding GCMs (see Supplementary Table S6). In the low MC group only, results revealed a significant linear time by regulation interaction (*b* = − 2.01, *SE* = 0.82, *t*(114) = − 2.54, *p* = 0.013), indicating that regulation affected cortisol trajectories of individuals with low MC, but not individuals with high MC while controlling for hormonal status. Specifically, individuals with low MC in the control compared to the reappraisal condition showed higher cortisol levels during recovery (+ 40 min: *t*(29) = 2.10, *p* = 0.045); though significance from post-hoc t-tests vanished when applying Bonferroni-correction. Thus, the control compared to the reappraisal condition resulted in higher cortisol responses in individuals with low MC, when controlling for hormonal status. Cortisol trajectories within the four groups are depicted in Fig. 1a.

#### Cortisol reactivity using the area under the curve with respect to increase (AUCi)

The 2 (MC) × 2 (regulation) ANOVA revealed no significant effect of either *MC* (*F*(1,87) = 0.93, *p* = 0.336, η_p_^2^ = 0.011), or regulation (*F*(1,87) = 2.11, *p* = 0.150, η_p_^2^ = 0.024), but a significant MC by regulation interaction effect (*F*(1,87) = 4.18, *p* = 0.044, η_p_^2^ = 0.046). Individuals with low MC showed stronger cortisol reactivity in the control compared to the reappraisal group (*F*(1,87) = 4.64,* p* = 0.034). No differences were found in the high MC group (*F*(1,87) = 0.26, *p* = 0.614). Results are reported excluding covariates, as sequential inclusion of covariates did not show any significant effects (*F*s <  = 1.93, *p*s >  = 0.131). Cortisol reactivity within the four groups is depicted in Fig. 1b.

### Effect of MC and regulation on subjective-emotional stress responses

#### Subjective-emotional stress trajectories using repeated measures

To explore an interaction between MC and regulation condition on subjective stress trajectories the basic model was extended by inclusion of regulation, MC and higher order interaction terms. Inclusion of none of these fixed effects increased model fit significantly (see Supplementary Table S4). Subjective stress trajectories within the four groups are depicted in Fig. 2a.

Similar results were observed for negative and positive affect ratings, that were each best explained by basic models with random intercepts, random slopes and fixed linear time trends (see Supplementary Tables S7–S9). Evaluation of the final basic model for negative affect, the linear trend of time (*b* = 0.13, *SE* = 0.02, *t*(185) = 6.17, *p* < 0.001) indicated that negative affect consistently increased over time (*p*s <  = 0.001, post-hoc t-tests). For positive affect, the linear time trend (*b* = 0.07, *SE* = 0.02, *t*(185) = 3.01, *p* = 0.003) indicates that positive affect only increased from prior to post TSST (between t = − 10 to t =  + 10, *p* = 0.046, post-hoc t-tests). Figure 2 depicts trajectories of negative (b) and positive (c) affect within the four groups.

In sum, subjective-emotional responses to stress revealed independent of MC and regulation condition. These results did not change when applying robust repeated-measures ANOVAs on 20% trimmed means for stress and negative affect ratings with groups as between-subjects, and time as within-subjects factor.

#### Subjective-emotional stress reactivity using the AUCi

Multiple 2 (MC) × 2 (regulation) ANOVAs did not show significant main or interaction effects on stress, negative and positive affect reactivity, each operationalized by the respective AUCi (*F*s <  = 2.12, *p*s >  = 0.149). Results did not change when applying corresponding robust ANOVAs on 20% trimmed means to analyze stress and negative affect reactivity. Results on subjective-emotional stress reactivity are summarized in Supplementary Table S10.

## Discussion

The primary goal of this study was to investigate whether ELA and cognitive reappraisal as an adaptive regulation strategy interact in affecting cortisol responses to psychosocial stress. For this, women with high or low early-life MC received either a regulation instruction asking them to reappraise or to focus on their senses during the subsequent TSST, while salivary cortisol levels were assessed repeatedly. Main analyses showed that the effect of instructed emotion regulation on cortisol stress responses depended on the extent of MC, while controlling for hormonal status. Focusing on senses compared to using reappraisal resulted in higher cortisol responses in women with low MC. In sum, these findings (1) confirm an interaction between MC and regulation condition; however, they (2) do not confirm the assumed direction, indicating that instead of reappraisal it was focusing on senses (designed as our control condition) that was related to increased cortisol stress responses in individuals with low MC.

To the best of our knowledge, this is the first experimental study to demonstrate an interaction between MC and a cognitive reappraisal condition with respect to cortisol stress responses. First indirect evidence has arisen from both ELA and ER research^46,62^, indicating heterogeneous results concerning the effects on HPA axis reactivity. Meta-analyses in both fields have thereby not only highlighted the role of moderating factors in general but have specifically suggested to consider ELA together with instructed ER in future studies^47^. Further empirical evidence stems from prior correlational work^54,55, 63^. These studies showed an interplay between ELA and overall ER capacity^54^, or specifically the habitual use of reappraisal^55^, that predicted cortisol responses to psychosocial stress. Finally, our finding aligns with theoretical models that assume that the effect of ER (strategies) depends on personality characteristics^56,64^. In the current study, we identified personal experiences, such as ELA, as another factor that interacts with instructed reappraisal in predicting cortisol stress reactivity. This finding could be observed when controlling for the effect of hormonal status. Menstrual cycle phase and oral contraceptive use (both affecting sex steroid hormones) are well-known impact factors of cortisol stress responses^65,66^**.** Specifically, women in the luteal compared to the follicular phase usually show higher salivary cortisol responses, with women using oral contraceptives showing blunted responses. Accordingly, the current results support more reduced cortisol responses in women using oral contraceptives, compared to naturally cycling females. This again highlights the relevance to assess hormonal status as a potential confounder in neuroendocrine research (e.g.,^67^).

With regard to the direction of the MC by regulation interaction the results surprisingly contradicted our initial hypothesis (i.e., higher cortisol responses following reappraisal in individuals with low MC). We next consider possible avenues on how to interpret these findings.

First, our results might imply that exposure to ELA is rather linked to HPA axis *hyper*reactivity, and that cognitive reappraisal successfully decreases exaggerated cortisol responses. Theoretical models in the field of ELA propose that environmental conditions early in life contribute to the development of different patterns of stress reactivity^22,68^. Threatening or unpredictable early-life environments, characterizing severe ELA^69^, are considered to favor exaggerated HPA axis responsivity. Empirical studies partially supported this assumption (albeit with inconsistent results), showing that ELA (i.e., lower early-life MC) was related to exaggerated cortisol responses to psychosocial stress^29,30^. Furthermore, cognitive reappraisal has been theorized to effectively regulate psychophysiological responses to emotion eliciting situations^36,37^. Empirically, reappraisal has been shown to reduce HPA axis responses to acute stress^70,71^. For instance, individuals who participated in a cognitive-behavioral training, including stress-reducing techniques (e.g., cognitive restructuring), compared to no training showed reduced cortisol responses to the TSST^71^. Although this comparison is limited due to duration and multicomponent nature of the training. A possible explanation why the effect of reappraisal differs between ELA groups is changes in the underlying cerebral mechanisms. The PFC has been identified as a central brain structure that is both involved in cognitive reappraisal^72^ and HPA axis regulation^73^, and affected by early-life stress^74^. For instance, the use of cognitive reappraisal to reinterpret negative emotional stimuli has been related to increased prefrontal brain activation^57,58^. Neuroimaging studies have also linked higher prefrontal activation during psychosocial stress with decreased cortisol responses^60,75^. Exposure to ELA particularly during sensitive periods of brain development contributes to an array of structural and/or functional alterations of the PFC^76,77^. Thus, implementing cognitive reappraisal might be related to altered patterns of prefrontal activation and downstream cortisol reactivity in individuals with high ELA.

Second, the results could indicate that exposure to ELA is linked to persistent HPA axis *hypo*responsivity, and that focusing on senses might provide an effective psychological intervention to increase such blunted cortisol stress responses. Various theoretical and empirical evidence suggests that HPA axis hyporeactivity develops as a consequence of exposure to ELA^5,62, 78^. Prolonged or repeated stress exposure is thereby considered to result in a long-term wear and tear of physiological stress systems, reflected in blunted patterns of stress reactivity^10^. In contrast, the effect of sensory focusing on HPA axis reactivity has not been studied in depth. Here, we chose sensory focus to provide a well-matched active control condition, considered superior to no-instruction control conditions (e.g.,^50^). Our results, however, imply that sensory focus might rather present another ER strategy. Indeed, it shows large overlap with attention deployment strategies^40,41^: The instruction resembled instructions for attention deployment, e.g., asking participants to attend or to focus on emotional experiences^40^; moreover, focusing on senses obviously requires individuals to deploy and control attention toward sensory impressions. Strategies to change the attentional focus are considered effective to regulate responses in emotional or stressful situations^36,37^. Accordingly, attentional trainings have been shown to alter acute HPA axis responses^79,80^. For instance, individuals who underwent an attentional control training, aiming to change attentional biases, showed higher cortisol reactivity when exposed to the TSST^80^. In line with this interpretation, neuronal mechanisms might again provide a suitable explanation for the diverging effect of sensory focus in individuals with high and low MC. The PFC has revealed to be involved not only in reappraisal but attentional control processes^81^. Since exposure to ELA impairs functional development of the PFC^77^, active attempts to control attention might be linked to different patterns of activation in individuals with high compared to low ELA, that subsequently affect downstream HPA axis responses. Thus, the PFC likewise constitutes a possible pathway to explain the effect of sensory focus, considered an attention deployment strategy, on cortisol responses in individuals with high ELA. In sum, this second explanation is possible, but less convincing from a purely descriptive perspective (see Fig. 1).

Third, our finding might also be explained by further differences between the low MC groups. To control for the effects of additional variables, we randomly assigned individuals to the regulation groups. Despite this, group differences might have existed and affected our results at hand.

Our exploratory analyses surprisingly did not confirm the robust finding that reappraisal decreases subjective-emotional stress responses (e.g.,^46^). However, methodological differences might contribute to this inconsistency. For instance, prior studies have often used passive emotion-induction paradigms (e.g., picture viewing), that induce comparably low and more easily controllable emotional responses. Reappraisal might be less effective in active stress induction paradigms (e.g., TSST), that trigger strong emotional reactions. In addition, reappraisal effects have often been reported with regard to specific emotions (e.g., anger). In the current study we referred to global composite measures of affect and stress, that might be too insensitive to reflect more specific effects of reappraisal.

This study underlies several central limitations. First, generalizability of our results is restricted to young, healthy women. We decided for a female sample to keep sex constant and comparable to prior work (e.g.,^51^). Future research should however aim for a more systematic evaluation of possible demographic impact factors such as sex or age^82^. A second limitation is that the degree of early-life MC was measured retrospectively via a self-report questionnaire. Retrospective, subjective measure may be biased for several reasons (e.g., memory effects, social desirability). Nevertheless, there is evidence that the subjective experience of adversity (e.g., as measured by self-report) rather than the pure objective exposure to it (e.g., official records) confers an increased health risk^83^. Third, we could not realize a fully balanced design as intended. Due to pandemic related obstacles in recruitment, final group sizes (*n*s ≥ 15) and, thus, power for statistical analyses was reduced. A post-hoc power analysis revealed a power of 48% to detect a MC by regulation interaction of medium effect size (mixed ANOVA, α = 0.05). Thus, we also re-analyzed cortisol data with MC as continuous variable, and found that the final model including MC, regulation condition and interactions with time, as well as hormonal status only showed a trend towards significance (*p* = 0.051; see Supplementary Table S11). Results and interpretation should therefore be considered preliminary before replicated in balanced samples. Fourth, our operationalization of ELA exclusively depends on the perception of early-life MC. Perceptual and caregiving aspects are central characteristics of ELA^20,84–86^. Since different types of ELA can exhibit diverging neuroendocrine effects^23^, our results may, however, not be generalizable to types other than MC. Fifth, interpretation of our results is limited due to the nature of the control instruction. With this active control task we intended (1) to reduce the use of habitual emotion regulation strategies, (2) to avoid waiting periods during the manipulation that might affect physiological outcomes, and (3) to design a well-matched control condition with similar attentional demands than reappraisal. Retrospectively, it remains unclear whether the instruction already affected cortisol trajectories and thus was not a suitable control condition that should not have instigated any regulation process^40^. Follow-up studies would benefit from the inclusion of a no-instruction control condition in terms of study comparability (e.g.,^50^) and interpretation of results. Finally, we want to emphasize the speculative character of our interpretation regarding biological mechanisms. We focus on the PFC and its central role in the interplay between ELA, emotion and stress regulation. However, other limbic areas, such as the hippocampus or amygdala, might similarly contribute to the observed findings. Thus, future neuroimaging studies are needed to gain a better understanding of underlying neuronal pathways.

To conclude, this study demonstrates the role of regulation strategies for HPA axis stress regulation in individuals with a history of ELA. Our findings indicate that focusing on one’s senses during a stressful task compared to cognitively reappraising the task results in increased cortisol stress responses of those individuals. Methodologically, these results imply the necessity to include and consider both ELA and ER measures in studies investigating HPA axis regulation. Moreover, they indicate cognitive ER as a promising target of psychological interventions aiming to restore HPA dysregulations in individuals with high ELA. Findings on decisive characteristics of cognitive ER, e.g., on the effect of acute stress on the ability to recruit cognitive ER^87^, also show its meaning and boundaries in everyday life. Since HPA axis dysregulation is one prominent mechanism linking ELA and psychopathology, understanding of effective treatment interventions can contribute to life-long health of individuals exposed to ELA.

## Methods

### Participants

Recruitment took place at the University and in the city of Konstanz using digital (e.g., social media) and analogue advertisement methods (e.g., flyer). Participants first completed an online screening questionnaire to determine study eligibility with respect to established exclusion criteria^67^. Those were an age younger than 18 or older than 30 years, current pregnancy, self-reported severe depressive symptoms (Beck’s Depression Inventory (BDI) II sum score > 28)^87,88^, under- or overweight (body mass index (BMI) < 17.5 or > 30), smoking > 5 cigarettes per day, working night-shifts, current drug or medication intake affecting the autonomous, endocrine or central nervous system and self-reported current mental or physical (cardiovascular, metabolic, endocrine) illness. Finally, we included female participants only, due to the well-known effect of sex^82^ and to keep the study comparable to prior work (e.g.,^51^). Moreover, the screening served to determine the extent of self-reported early-life MC using the Parental Bonding Instrument (PBI) (^89^, German version:^90^), based on which participants were allocated to either a high or low MC group (see below).

### Procedure

Eligible participants were invited to the laboratory sessions that took place at the Centre of Psychiatry in Konstanz (November 2020 to July 2021). Sessions were conducted in the afternoons between 03:00 and 06:30pm to control for circadian variations in cortisol secretion^91^ and lasted for approximately 1.5 h. Beforehand, participants were requested to refrain from consumption of food and (soft-)drinks containing theine or caffeine and smoking two hours prior to session start. They were further instructed to abstain from alcohol eighteen hours before and from intense physical exercise the day before the session^67^.

#### Study overview

**Figure 3:**
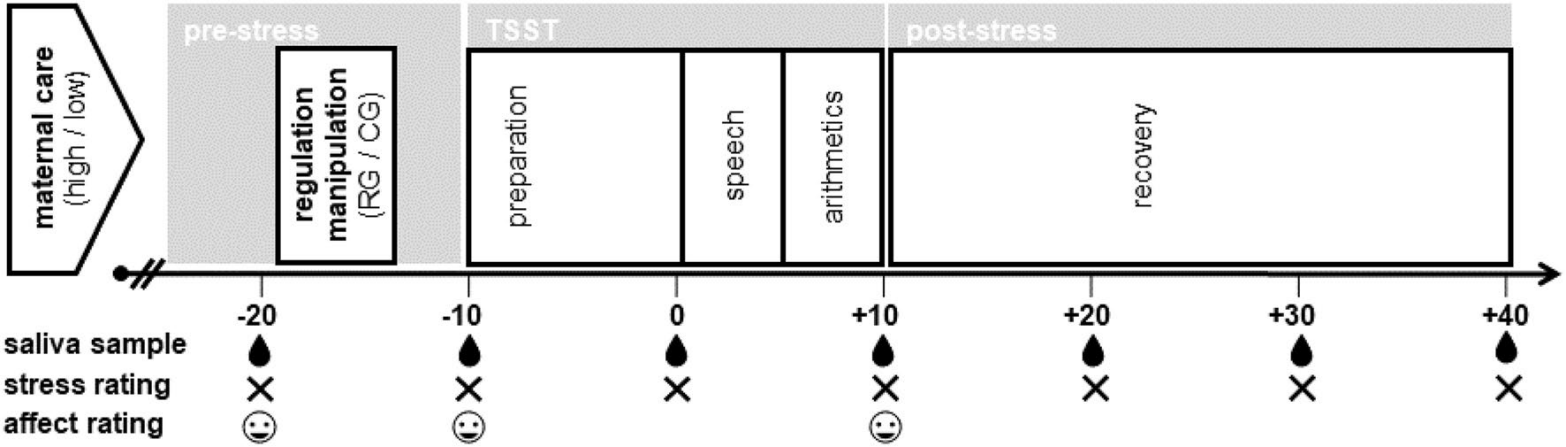
Overview of study procedure. The manipulation of emotion regulation required participants to either use cognitive reappraisal (RG) or to focus on senses (CG) during the TSST. TSST = Trier Social Stress Test, RG = reappraisal group, CG = control group.

Laboratory sessions consisted of a pre-stress phase (30 min), that served as an acclimatization and to provide regulation instructions, followed by a stress (20 min) and a final recovery phase (30 min) (see Fig. 3 for study overview).

Upon arrival, participants first provided written informed consent and were introduced to the saliva sampling. Following a set of questions (e.g., on menstrual cycle) and resting (5 min) to further acclimatize to the laboratory set-up^92^, a first saliva sample was collected (− 20 min). As part of the subsequent experimental manipulation (5 min), participants received written instructions to cognitively reappraise (RG) or to focus on their senses (CG) during a subsequent stress task (see below). During the stress phase, participants underwent the TSST^61^, consisting of a preparation (10 min) and stress period (10 min). In the post-stress recovery phase participants completed further trait questionnaires (e.g., on self-esteem, habitual emotion regulation). At the end of a session participants provided a final seventh saliva sample (+ 40 min), were debriefed and received financial compensation (15€) or course credit (1.5 h). The study was conducted according to the ethical principles of the Declaration of Helsinki and approved by the Ethics Committee of the University of Konstanz (IRB statement 12/2017).

#### Experimental manipulation

Material to manipulate emotion regulation referred to either cognitive reappraisal or human senses (see below). It each consisted of (1) a short text to introduce the respective topic, (2) a specific task instruction, and (3) an open-ended question asking participants to summarize provided texts and instructions which was intended to promote consolidation and check correct understanding (see manipulation check). Participants received 5 min to read the texts and instructions (1, 2) and to answer the question in writing (3). When completed within less than 5 min participants were asked to start over again reading. Material was designed based on provided scripted material^50,51^ and available recommendations^93^.

##### Reappraisal instruction

The text introduced participants to the meaning and rationale of cognitive reappraisal, such that re-interpreting a potentially stressful situation could help to change and positively influence one’s reaction to it. An example was provided to increase understanding and concrete application of reappraisal. The second part comprised the specific reappraisal instruction (modified version of^50^): Participants were asked to try to think of *positive aspects of the task* (e.g., lessons they might learn) during preparation of the following speech task. During the task they were requested to adopt a *neutral and objective attitude towards their performance*, considering it from a *third-person perspective,* as well as to consider the committee’s evaluation in an objective, analytical and *emotionally detached* manner.

##### Control instruction

Material comprised an initial short text about human senses, such as information about their number and function (modified version of^51^). It also included an example, e.g., on how proprioception can be experienced in daily life, to improve understanding. The second part included the specific task instruction: Participants were requested to focus on as many of their senses during performing the subsequent speech task as possible, such as room temperature, odors, visual and auditory perception of the committee. The overall length and complexity of material was considered comparable to the reappraisal instruction.

##### Manipulation check

Following the manipulation, participants’ understanding of the respective instruction was checked using two items. The first item provided an incorrect description of the respective instruction (RG: “I am to emotionally evaluate the situation from a third-person perspective”, CG: “I am to focus particularly on one of my senses during the situation.”); in contrast, the second item comprised a correct description giving key elements of the respective instruction (RG: “I am to reappraise the situation from a neutral and objective third-person perspective.”, CG: “I am to focus on as many of my senses as possible during the situation.”). Items had to be answered on a 5-point Likert scale ranging from 1 (*not at all*) to 5 (*very much*). Correct understanding was assumed for scores of 1 or 2 (item 1) and scores of 4 or 5 (item 2). Although such item-based manipulation checks are well-established in emotion regulation research (e.g.,^94,95^), they often lack psychometric validation and favor socially desirable responses. Qualitative measures, such as the use of open-ended questions has been recommended to address such shortcomings^93^. Thus, if answers to the manipulation check items indicated non-understanding, we examined the answer to the open-ended question as part of the manipulation material (see above). In case of a correct description, we assumed correct understanding of the manipulation instruction.

##### Stress induction

To induce psychosocial stress the TSST^61^ was applied. As the most prominently used standardized stress paradigm the TSST has been shown to reliably induces neuroendocrine and subjective-emotional responses^96,97^. It consists of a preparation (10 min) and subsequent test period, including a speech task (5 min) and mental arithmetic tasks (5 min). During the preparation period participants were instructed and prepared for a mock job interview (speech task); in the current study, they additionally received a written summary of the regulation instruction provided beforehand, to re-read it at the end of the preparation phase. For the test period participants were brought to an adjacent room to perform the speech and the subsequent mental arithmetic task (counting back from 1022 in steps of 13 aloud). Tasks were video recorded if participants did not contradict in advance and conducted in front of a confederate, white-coated committee (one female, one male). After completion of the stress tasks, participants were guided back to the preparation room for the recovery.

### Measures

#### Neuroendocrine measure

Cortisol levels were determined from seven saliva samples that were collected at pre-defined timepoint (− 20, − 10, 0, + 10, + 20, + 30, + 40 min) using Salivettes (Sarstedt, Nümbrecht, Germany) (see Fig. 3). Samples were stored at − 20 °C before they were thawed for analyses at the biochemical laboratory of the University of Konstanz. For the detection of cortisol levels (nmol/l) samples were centrifugated at 2500 rpm for 10 min and analyzed in duplicate using a commercially available competitive enzyme immunosorbent assay (Cortisol Saliva ELISA, RE-52611, IBL International GmbH, Hamburg, Germany), a reliable method to assess free salivary cortisol^98^. Mean inter- and intra-assay coefficients of variance were 6.6% and 7.9%, respectively.

#### Psychological measures

##### Maternal care

The Parental Bonding Instrument (PBI) (^89^; German version:^90^) retrospectively assesses the extent of perceived parental care and overprotection during the first 16 years of life by self-report. The MC subscale comprises 12 items to be answered on a 4-point Likert scale ranging from 0 (*very unlike*) to 3 (*very like*). A higher MC sum score (range: 0–36, cut off value: 27) indicates higher perceived affectionate and emotionally warm maternal care. The MC subscale was used to determine the extent of early-life MC as part of the online screening and to categorize participants to either a high MC (score > 27) or low MC group (score <  = 27)^89^.

##### Subjective stress

The Affect Grid (AG) (99) was used as a single-item measures to repeatedly assess current affect. Scores for the two dimensions pleasure and arousal (ranges: 1–9), built up a composite stress score that ranges from 1 to 81^100^. Higher scores indicate a higher degree of subjective stress. The AG was administered seven times along with saliva sampling to capture the subjective stress response to stress (see Fig. 3).

##### Affect

The Positive and Negative Affect Schedule (PANAS) (^101^; German version:^102^) was administered to assess self-reported positive and negative affect. The subscales Negative and Positive Affect each consist of ten items that are answered on a 5-point Likert scale ranging from 1 (*very slightly or not at all*) to 5 (*very much*). Higher sum scores for each subscale (range: 10–50) indicate higher levels of negative and positive affect, respectively. The PANAS was answered three times (pre-manipulation, pre- and post-TSST) to capture changes in affect (see Fig. 3).

##### Covariates

Variables that may affect neuroendocrine responses^67,82^ were assessed additionally to evaluate their impact as possible covariates. Multiple questions were used to measure age, BMI, and hormonal status by self-report. Hormonal status (contraceptive intake, luteal phase, follicular phase (e.g.,^103^), unclear phase) was calculated from reported oral contraceptive intake, duration and onset of last menstrual cycle^67^. The last category was added to account for individuals who reported unregular or extended cycle duration (> 37 days,^104^), or who did not report the exact onset of last menses. Questionnaires were applied to assess symptoms of depression (BDI-II) (^87^; German version:^105^), self-esteem (Rosenberg Self-Esteem Scale (RSES)) (^106^; German version:^107^), and habitual use of emotion regulation strategies (Emotion Regulation Questionnaires (ERQ)) (^39^; German version:^108^). A complete list of psychological measures that were assessed but not analyzed within this work is provided on the Open Science Framework (https://osf.io/6bfhx/).

### Design and sample size

A between-subjects 2 MC (high, low) × 2 regulation (reappraisal, control) study design resulting in four distinct groups was realized. Participants were first quasi-experimentally assigned to either a low or high MC group; within each group they were then alternately assigned to the reappraisal (RG) or control group (CG) as introduced before.

Sample size was determined a priori using G*Power 3.1^109^. Based on a 2 (MC) × 2 (regulation) factorial ANOVA (1-β = 0.8, α = 0.05) calculations suggested a sample of *N* = 128 to detect a MC by regulation interaction of moderate effect size (*η*_*p*_^*2*^ = 0.06). Due to pandemic related testing restrictions and time restraints of the associated personnel we were able to collect data of *N* = 97 participants only. Six participants had to be excluded retrospectively as (1) they did not fulfill inclusion criteria (*n* = 3), (2) did not understand the manipulation instruction (*n* = 1), or (3) had completely missing cortisol levels due to too little amount of provided saliva (*n* = 2). Thus, statistical analyses were based on *N* = 93, or *N* = 91 (analyses on cortisol).

### Statistical analyses

Analyses were performed using R statistical software (version 4.0.4; R Core Team, 2021) and RStudio (version 2022.2.3.492; Rstudio Team, 2022), the packages *nlme*^110^, *WRS2*^111^, and *ggplot2*^112^. Preprocessing of data (cortisol, stress and affect ratings) first included screening for and replacement of missing values as needed. Second, statistical outliers were winsorized at each timepoint, replacing them with the sample’s mean ± 3 SD at the respective timepoint. Third, natural log transformation of cortisol levels was applied to correct for skewness of data. Finally, we calculated the area under the curve with respect to increase (AUCi, Pruessner et al.^113^), as a cumulative measure of overall stress reactivity for cortisol (AUCi_cort_, within 0 to + 40 min), subjective stress (AUCi_stress_, within − 20 to + 40 min) and affect (AUCi_NA_ (negative affect) resp. AUCi_PA_ (positive affect), within − 20 to + 10 min). Natural log transformation of (AUCi_cort_ + 340) was applied to correct for skewness. Details on data preprocessing are provided in Supplementary S1.

As first part of the analyses we tested whether group differences occurred with regard to potential covariates. Multiple one-way Analyses of Variance (ANOVAs) or Fisher’s exact test were conducted with group as independent variable and age, BMI, depressiveness, self-esteem, trait ER strategies (suppression, reappraisal) and hormonal status as dependent variables.

Second, two manipulation checks were conducted. To check participants’ understanding of the regulation instructions, two manipulation check items were examined descriptively. Group differences in understanding were tested using multiple Kruskal–Wallis Tests with scores of item 1 or item 2 as dependent, and the four groups as independent variables. To check the success of the TSST to induce stress, we used multilevel GCMs (see below).

Next, to test the hypothesized interaction of MC and regulation condition on cortisol stress responses we followed a two-way approach^100,114^, considering both (1) cortisol trajectories and (2) overall cortisol reactivity. As we were interested in stress-induced responses we used baseline(0 min)-adjusted cortisol levels starting from stressor onset (0 to + 40 min). Concerning cortisol trajectories, the effects were tested using GCMs within the multilevel framework. A basic model was built to describe changes in cortisol levels as a function of time (fixed effect), while accounting for interindividual variation (random effects); followed by an interaction model, that included MC, regulation condition and interaction terms, as well as possible covariates as fixed effects^115,116^. The basic model served to check successful stress induction; the interaction model was used to test the hypothesized MC by regulation by time interaction. Models were built by step-wise inclusion of effects based on best fit: fixed intercept, random intercepts (across individuals), fixed time trends (linear, quadratic, cubic), random time trends (linear, quadratic, cubic) and a first-order autoregressive covariance structure (AR) were included to build the basic model; next, regulation, regulation by time, MC, MC by time, regulation by MC and the MC by regulation by time interaction were included as fixed effects to build the interaction model, followed by inclusion of possible covariates and covariates by time interactions. Thereby, each covariate was added separately and, after evaluation of the model fit, was excluded to repeat the procedure for the next covariate. MC (levels: low MC = 0, high MC = 1), regulation condition (levels: control = 0, reappraisal = 1) and hormonal status (levels: oral contraceptives = 0, follicular phase = 1, luteal phase = 2, unclear phase = 3) were entered as factors into models. Models of increasing complexity were compared pairwise, and changes in model fit were assessed by means of the log-likelihood ratio and ANOVAs. Final models with best model fit are evaluated with regression coefficients being reported.

Concerning cortisol reactivity, a 2 (MC) × 2 (regulation) AN(C)OVA was performed with the AUCi_cort_ as dependent variable. A significant interaction was followed up by simple-effect analysis.

Finally, we explored the effects of MC and regulation condition on subjective-emotional stress responses (stress, negative and positive affect). Effects were similarly analyzed with regard to (1) changes in subjective-emotional levels over time using GCMs, and (2) subjective-emotional reactivity (AUCi_stress,_ AUCi_NA_ and AUCi_PA_) using multiple ANOVAs. Analyses were complimented by robust ANOVAS on 20% trimmed means for stress and negative affect, due to violation of parametric test assumptions.

Across analyses the level of significance was set to α = 0.05. Significant effects were followed up by Bonferroni-corrected post-hoc t-tests. Model assumptions were checked visually or test statistically. Normal distribution was assessed using Shapiro–Wilk, homogeneity of variance was assessed using Levene’s tests.

### Supplementary Information

Supplementary Information.

## Data Availability

Data and code for statistical analyses are available at https://osf.io/6bfhx/ (Open Science Framework, Project DOI: https://doi.org/10.17605/OSF.IO/6BFHX).

## References

[1] Felitti VJ (1998). Relationship of childhood abuse and household dysfunction to many of the leading causes of death in adults: The Adverse Childhood Experiences (ACE) Study. Am. J. Prev. Med..

[2] McCrory C, Dooley C, Layte R, Kenny RA (2015). The lasting legacy of childhood adversity for disease risk in later life. Health Psychol..

[3] Lovallo WR (2013). Early life adversity reduces stress reactivity and enhances impulsive behavior: Implications for health behaviors. Int. J. Psychophysiol..

[4] Lupien SJ, McEwen BS, Gunnar MR, Heim C (2009). Effects of stress throughout the lifespan on the brain, behaviour and cognition. Nat. Rev..

[5] McEwen BS (1998). Stress, adaptation, and disease: Allostasis and allostatic load. Ann. N. Y. Acad. Sci..

[6] Kiecolt-Glaser JK, Renna ME, Shrout MR, Madison AA (2020). Stress reactivity: What pushes us higher, faster, and longer—and why it matters. Curr. Dir. Psychol. Sci..

[7] Sapolsky RM, Romero LM, Munck AU (2000). How do glucocorticoids influence stress responses? Integrating permissive, suppressive, stimulatory, and preparative actions. Endocr. Rev..

[8] Chrousos GP (2009). Stress and disorders of the stress system. Nat. Rev. Endocrinol..

[9] Turner AI (2020). Psychological stress reactivity and future health and disease outcomes: A systematic review of prospective evidence. Psychoneuroendocrinology.

[10] McEwen BS (2000). Allostasis and allostatic load: Implications for neuropsychopharmacology. Neuropsychopharmacology.

[11] McEwen BS, Gianaros PJ (2010). Central role of the brain in stress and adaptation: Links to socioeconomic status, health, and disease. Ann. N. Y. Acad. Sci..

[12] Agorastos A, Pervanidou P, Chrousos GP, Kolaitis G (2018). Early life stress and trauma: Developmental neuroendocrine aspects of prolonged stress system dysregulation. Hormones.

[13] Kalmakis KA, Chandler GE (2014). Adverse childhood experiences: Towards a clear conceptual meaning. J. Adv. Nurs..

[14] Lai CLJ, Lee DYH, Leung MOY (2020). Childhood adversities and salivary cortisol responses to the Trier Social Stress Test: A systematic review of studies using the Children Trauma Questionnaire (CTQ). Int. J. Environ. Res. Public Health.

[15] Bugental DB, Martorell GA, Barraza V (2003). The hormonal costs of subtle forms of infant maltreatment. Horm. Behav..

[16] Oosterman M, de Schipper JC, Fisher PA, Dozier M, Schuengel C (2010). Autonomic reactivity in relation to attachment and early adversity among foster children. Dev. Psychopathol..

[17] Chen Y, Baram TZ (2016). Toward understanding how early-life stress reprograms cognitive and emotional brain networks. Neuropsychopharmacology.

[18] Petrullo LA, Mandalaywala TM, Parker KJ, Maestripieri D, Higham JP (2016). Effects of early life adversity on cortisol/salivary alpha-amylase symmetry in free-ranging juvenile rhesus macaques. Horm. Behav..

[19] BosquetEnlow M (2014). Maternal sensitivity and infant autonomic and endocrine stress responses. Early. Hum. Dev..

[20] Vergara-Lopez C, Chaudoir S, Bublitz M, O'Reilly Treter M, Stroud L (2016). The influence of maternal care and overprotection on youth adrenocortical stress response: a multiphase growth curve analysis. Stress.

[21] Strüber N, Strüber D, Roth G (2014). Impact of early adversity on glucocorticoid regulation and later mental disorders. Neurosci. Biobehav. Rev..

[22] Del Giudice M, Ellis BJ, Shirtcliff EA (2011). The adaptive calibration model of stress responsivity. Neurosci. Biobehav. Rev..

[23] Hosseini-Kamkar N, Lowe C, Morton JB (2021). The differential calibration of the HPA axis as a function of trauma versus adversity: A systematic review and p-curve meta-analyses. Neurosci. Biobehav. Rev..

[24] Voellmin A (2015). Blunted endocrine and cardiovascular reactivity in young healthy women reporting a history of childhood adversity. Psychoneuroendocrinology.

[25] Young ES (2021). Life stress and cortisol reactivity: An exploratory analysis of the effects of stress exposure across life on HPA-axis functioning. Dev. Psychopathol..

[26] Heim C (2000). Pituitary-adrenal and autonomic responses to stress in women after sexual and physical abuse in childhood. Jama.

[27] Rao U, Hammen C, Ortiz LR, Chen L-A, Poland RE (2008). Effects of early and recent adverse experiences on adrenal response to psychosocial stress in depressed adolescents. Biol. Psychiatry.

[28] DeSantis SM (2011). Gender differences in the effect of early life trauma on hypothalamic-pituitary-adrenal axis functioning. Depress. Anxiety..

[29] Engert V (2010). Perceived early-life maternal care and the cortisol response to repeated psychosocial stress. J. Psychiatry Neurosci..

[30] Pruessner JC, Champagne F, Meaney MJ, Dagher A (2004). Dopamine release in response to a psychological stress in humans and its relationship to early life maternal care: a positron emission tomography Study Using [11C]Raclopride. J. Neurosci..

[31] Rab SL, Admon R (2021). Parsing inter- and intra-individual variability in key nervous system mechanisms of stress responsivity and across functional domains. Neurosci. Biobehav. Rev..

[32] Reilly EB, Gunnar MR (2019). Neglect, HPA axis reactivity, and development. Int. J. Dev. Neurosci..

[33] Gross JJ, Thompson RA, Gross JJ (2007). Handbook of emotion regulation.

[34] Naragon-Gainey K, McMahon TP, Chacko TP (2017). The structure of common emotion regulation strategies: A meta-analytic examination. Psychol. Bull..

[35] Gross JJ (2015). Emotion regulation: Current status and future prospects. Psychol. Inq..

[36] Gross JJ (1998). Antecedent-and response-focused emotion regulation: Divergent consequences for experience, expression, and physiology. J. Pers. Soc. Psychol..

[37] Gross JJ (2002). Emotion regulation: Affective, cognitive, and social consequences. Psychophysiology.

[38] Lazarus RS, Folkman S (1987). Transactional theory and research on emotions and coping. Eur. J. Pers..

[39] Gross JJ, John OP (2003). Individual differences in two emotion regulation processes: Implications for affect, relationships, and well-being. J. Pers. Soc. Psychol..

[40] Webb TL, Miles E, Sheeran P (2012). Dealing with feeling: a meta-analysis of the effectiveness of strategies derived from the process model of emotion regulation. Psychol. Bull..

[41] Zähringer J, Jennen-Steinmetz C, Schmahl C, Ende G, Paret C (2020). Psychophysiological effects of downregulating negative emotions: Insights from a meta-analysis of healthy adults. Front. Psychol..

[42] Gross JJ (2013). Emotion regulation: Taking stock and moving forward. Emotion.

[43] McRae K (2016). Cognitive emotion regulation: A review of theory and scientific findings. Curr. Opin. Behav. Sci..

[44] Miu AC (2022). Emotion regulation as mediator between childhood adversity and psychopathology: A meta-analysis. Clin. Psychol. Rev..

[45] Riepenhausen A (2022). Positive cognitive reappraisal in stress resilience, mental health, and well-being: A comprehensive systematic review. Emot. Rev..

[46] Liu JJW, Ein N, Gervasio J, Vickers K (2019). The efficacy of stress reappraisal interventions on stress responsivity: A meta-analysis and systematic review of existing evidence. PloS one.

[47] Mikkelsen MB, Tramm G, Zachariae R, Gravholt CH, O’Toole MS (2021). A systematic review and meta-analysis of the effect of emotion regulation on cortisol. Compr. Psychoneuroendocrinol..

[48] Lam S, Dickerson SS, Zoccola PM, Zaldivar F (2009). Emotion regulation and cortisol reactivity to a social-evaluative speech task. Psychoneuroendocrinology.

[49] Raymond C, Marin M-F, Juster R-P, Lupien SJ (2019). Should we suppress or reappraise our stress?: The moderating role of reappraisal on cortisol reactivity and recovery in healthy adults. Anxiety Stress Coping.

[50] Denson TF, Creswell JD, Terides MD, Blundell K (2014). Cognitive reappraisal increases neuroendocrine reactivity to acute social stress and physical pain. Psychoneuroendocrinology.

[51] Jentsch VL, Wolf OT (2020). The impact of emotion regulation on cardiovascular, neuroendocrine and psychological stress responses. Biol. Psychol..

[52] Mauersberger H, Hoppe A, Brockmann G, Hess U (2018). Only reappraisers profit from reappraisal instructions: Effects of instructed and habitual reappraisal on stress responses during interpersonal conflicts. Psychophysiology.

[53] Kirlic N, Cohen ZP, Singh MK (2020). Is there an ace up our sleeve? A review of interventions and strategies for addressing behavioral and neurobiological effects of adverse childhood Experiences in Youth. Advers. Resilience Sci..

[54] England-Mason G (2017). Difficulties with emotion regulation moderate the association between childhood history of maltreatment and cortisol reactivity to psychosocial challenge in postpartum women. Horm. Behav..

[55] Johnson AE, Perry NB, Hostinar CE, Gunnar MR (2019). Cognitive-affective strategies and cortisol stress reactivity in children and adolescents: Normative development and effects of early life stress. Dev. Psychobiol..

[56] Kobylińska D, Kusev P (2019). Flexible emotion regulation: How situational demands and individual differences influence the effectiveness of regulatory strategies. Front. Psychol..

[57] McRae K (2010). The neural bases of distraction and reappraisal. J. Cogn. Neurosci..

[58] Nelson BD, Fitzgerald DA, Klumpp H, Shankman SA, Phan KL (2015). Prefrontal engagement by cognitive reappraisal of negative faces. Behav. Brain. Res..

[59] Danese A, McEwen BS (2012). Adverse childhood experiences, allostasis, allostatic load, and age-related disease. Physiol. Behav..

[60] Zhong X (2019). Childhood maltreatment experience influences neural response to psychosocial stress in adults: An fMRI study. Front. Psychol..

[61] Kirschbaum C, Pirke K-M, Hellhammer DH (1993). The ‘Trier Social Stress Test’—A tool for investigating psychobiological stress responses in a laboratory setting. Neuropsychobiology.

[62] Bunea IM, Szentágotai-Tătar A, Miu AC (2017). Early-life adversity and cortisol response to social stress: A meta-analysis. Transl. Psychiatry.

[63] Perry NB, Donzella B, Parenteau AM, Desjardins C, Gunnar MR (2019). Emotion regulation and cortisol reactivity during a social evaluative stressor: A study of post-institutionalized youth. Dev. Psychobiol..

[64] Troy AA, Shallcross AJ, Mauss IB (2016). Corrigendum: A person-by-situation approach to emotion regulation: Cognitive reappraisal can either help or hurt, depending on the context. Psychol. Sci..

[65] Gervasio J, Zheng S, Skrotzki C, Pachete A (2022). The effect of oral contraceptive use on cortisol reactivity to the Trier Social Stress Test: A meta-analysis. Psychoneuroendocrinology.

[66] Montero-López E (2018). The relationship between the menstrual cycle and cortisol secretion: Daily and stress-invoked cortisol patterns. Int. J. Psychophysiol..

[67] Strahler J, Skoluda N, Kappert MB, Nater UM (2017). Simultaneous measurement of salivary cortisol and alpha-amylase: Application and recommendations. Neurosci. Biobehav. Rev..

[68] Boyce WT, Ellis BJ (2005). Biological sensitivity to context: I. An evolutionary-developmental theory of the origins and functions of stress reactivity. Dev. Psychopathol..

[69] Berman IS (2022). Measuring early life adversity: A dimensional approach. Dev. Psychopathol..

[70] Carlson JM, Dikecligil GN, Greenberg T, Mujica-Parodi LR (2012). Trait reappraisal is associated with resilience to acute psychological stress. J. Res. Pers..

[71] Gaab J (2003). Randomized controlled evaluation of the effects of cognitive–behavioral stress management on cortisol responses to acute stress in healthy subjects. Psychoneuroendocrinology.

[72] Morawetz C, Bode S, Derntl B, Heekeren HR (2017). The effect of strategies, goals and stimulus material on the neural mechanisms of emotion regulation: A meta-analysis of fMRI studies. Neurosci. Biobehav. Rev..

[73] Pruessner JC (2010). Stress regulation in the central nervous system: Evidence from structural and functional neuroimaging studies in human populations—2008 Curt Richter Award Winner. Psychoneuroendocrinology.

[74] Dedovic K, Duchesne A, Andrews J, Engert V, Pruessner JC (2009). The brain and the stress axis: The neural correlates of cortisol regulation in response to stress. NeuroImage.

[75] Harrewijn A (2020). Associations between brain activity and endogenous and exogenous cortisol—A systematic review. Psychoneuroendocrinology.

[76] Burrus C (2013). Developmental trajectories of abuse—An hypothesis for the effects of early childhood maltreatment on dorsolateral prefrontal cortical development. Med. Hypotheses.

[77] Heim C, Entringer S, Buss C (2019). Translating basic research knowledge on the biological embedding of early-life stress into novel approaches for the developmental programming of lifelong health. Psychoneuroendocrinology.

[78] Fries E, Hesse J, Hellhammer J, Hellhammer DH (2005). A new view on hypocortisolism. Psychoneuroendocrinology.

[79] Dandeneau SD, Baldwin MW, Baccus JR, Sakellaropoulo M, Pruessner JC (2007). Cutting stress off at the pass: Reducing vigilance and responsiveness to social threat by manipulating attention. J. Pers. Soc. Psychol..

[80] Pilgrim K, Ellenbogen MA, Paquin K (2014). The impact of attentional training on the salivary cortisol and alpha amylase response to psychosocial stress: Importance of attentional control. Psychoneuroendocrinology.

[81] Wang L (2010). Effective connectivity of the fronto-parietal network during attentional control. J. Cogn. Neurosci..

[82] Zänkert S, Bellingrath S, Wüst S, Kudielka BM (2019). HPA axis responses to psychological challenge linking stress and disease: What do we know on sources of intra- and interindividual variability?. Psychoneuroendocrinology.

[83] Danese A, Widom CS (2020). Objective and subjective experiences of child maltreatment and their relationships with psychopathology. Nat. Human Behav..

[84] Loman MM, Gunnar MR (2010). Early experience and the development of stress reactivity and regulation in children. Neurosci. Biobehav. Rev..

[85] Pollak SD, Smith KE (2021). Thinking clearly about biology and childhood adversity: Next steps for continued progress. Perspect. Psychol. Sci..

[86] Smith KE, Pollak SD (2021). Rethinking concepts and categories for understanding the neurodevelopmental effects of childhood adversity. Perspect. Psychol. Sci..

[87] Beck, A. T., Steer, R. A. & Brown, G. K. *Beck Depression Inventory-II (BDI-II)* (The Psychological Corporation, 1996).

[88] Kühner C, Bürger C, Keller F, Hautzinger M (2007). Reliabilität und Validität des revidierten Beck-Depressionsinventars (BDI-II). Befunde aus deutschsprachigen Stichproben. Der Nervenarzt.

[89] Parker G, Tupling H, Brown LB (1979). A parental bonding instrument. Br. J. Med. Psychol..

[90] Benz ABE (2022). Psychometrische Kennwerte einer deutschen Übersetzung des Parental Bonding Instrument. Psychother. Psychosom. Med. Psychol..

[91] Miller R (2016). The CIRCORT database: Reference ranges and seasonal changes in diurnal salivary cortisol derived from a meta-dataset comprised of 15 field studies. Psychoneuroendocrinology.

[92] Balodis IM, Wynne-Edwards KE, Olmstead MC (2010). The other side of the curve: examining the relationship between pre-stressor physiological responses and stress reactivity. Psychoneuroendocrinology.

[93] Aldao A (2013). The future of emotion regulation research: Capturing context. Perspect. Psychol. Sci..

[94] Campbell-Sills L, Barlow DH, Brown TA, Hofmann SG (2006). Effects of suppression and acceptance on emotional responses of individuals with anxiety and mood disorders. Behav. Res. Ther..

[95] Wolgast M, Lundh L-G, Viborg G (2011). Cognitive reappraisal and acceptance: an experimental comparison of two emotion regulation strategies. Behav. Res. Ther..

[96] Allen AP, Kennedy PJ, Cryan JF, Dinan TG, Clarke G (2014). Biological and psychological markers of stress in humans: Focus on the Trier Social Stress Test. Neurosci. Biobehav. Rev..

[97] Het S, Rohleder N, Schoofs D, Kirschbaum C, Wolf OT (2009). Neuroendocrine and psychometric evaluation of a placebo version of the ‘Trier Social Stress Test’. Psychoneuroendocrinology.

[98] Dressendörfer RA, Kirschbaum C, Rohde W, Stahl F, Strasburger CJ (1992). Synthesis of a cortisol-biotin conjugate and evaluation as a tracer in an immunoassay for salivary cortisol measurement. J. Steroid Biochem. Mol. Biol..

[99] Russell JA, Weiss A, Mendelsohn GA (1989). Affect Grid: A single-item scale of pleasure and arousal. J. Pers. Soc. Psychol..

[100] Meier M (2021). Effects of psychological, sensory, and metabolic energy prime manipulation on the acute endocrine stress response in fasted women. Psychoneuroendocrinology.

[101] Watson D, Clark LA, Tellegen A (1988). Development and validation of brief measures of positive and negative affect: The PANAS scales. J. Pers. Soc. Psychol..

[102] Krohne HW, Egloff B, Kohlmann CW, Tausch A (1996). Investigations with a German version of the positive and negative affect schedule (PANAS). Diagnostica.

[103] Kirschbaum C, Kudielka BM, Gaab J, Schommer NC, Hellhammer DH (1999). Impact of gender, menstrual cycle phase, and oral contraceptives on the activity of the hypothalamus-pituitary-adrenal axis. Psychosom. Med..

[104] Schmalenberger KM (2021). How to study the menstrual cycle: Practical tools and recommendations. Psychoneuroendocrinology.

[105] Hautzinger, M., Keller, F. & Kühner, C. *Beck Depressions-Inventar (BDI-II). Revision.* (Harcourt Test Services., 2006).

[106] Rosenberg M (1965). Society and the adolescent self-image.

[107] von Collani G, Herzberg PY (2003). Zur internen Struktur des globalen Selbstwertgefühls nach Rosenberg. Zeitschrift für Differentielle und Diagnostische Psychologie.

[108] Abler B, Kessler H (2009). Emotion Regulation Questionnaire - eine deutschsprachige Fassung des ERQ von Gross und John. Diagnostica.

[109] Faul F, Erdfelder E, Buchner A, Lang A-G (2009). Statistical power analyses using G*Power 3.1: Tests for correlation and regression analyses. Behav. Res. Methods.

[110] Pinheiro, J., Bates, D., DebRoy, S. & Sarkar, D. nlme: Linear and Nonlinear Mixed Effects Models (2021).

[111] Mair P, Wilcox R (2020). Robust statistical methods in R using the WRS2 package. Behav. Res. Methods.

[112] Wickham H (2016). ggplot2: Elegant Graphics for Data Analysis.

[113] Pruessner JC, Kirschbaum C, Meinlschmid G, Hellhammer DH (2003). Two formulas for computation of the area under the curve represent measures of total hormone concentration versus time-dependent change. Psychoneuroendocrinology.

[114] Bentele UU (2021). The impact of maternal care and blood glucose availability on the cortisol stress response in fasted women. J. Neural Transm..

[115] Bliese PD, Ployhart RE (2002). Growth modeling using random coefficient models: Model building, testing, and illustrations. Organ. Res. Methods.

[116] Curran PJ, Obeidat K, Losardo D (2010). Twelve frequently asked questions about growth curve modeling. J. Cogn. Dev..

