# Supplementary material for: The effect of cognitive reappraisal and early-life maternal care on neuroendocrine stress responses
Source: Sci Rep. 2024 Mar 21;14:6837. doi: 10.1038/s41598-024-57106-x (PMC10957921; doi:10.1038/s41598-024-57106-x)
Supplement: Supplementary file 1 — Supplementary Information. [file 41598_2024_57106_MOESM1_ESM.pdf]

Supplementary Material

The effect of cognitive reappraisal and early-life maternal care on neuroendocrine stress responses

Ulrike U. Bentele*[1], Elea S. C. Klink[1], Annika B. E. Benz[1], Maria Meier[1,3], Raphaela J. Gaertner[1], Bernadette F. Denk[1,2], Stephanie J. Dimitroff[4], Eva Unternaehrer[1,3], & Jens C. Pruessner[1,2]

[1]*Department of Psychology, Division of Neuropsychology, University of Konstanz, Constance, Germany*

[2]*Centre for the Advanced Study of Collective Behaviour, University of Konstanz, Constance, Germany*

[3]*Child- and Adolescent Research Department, University Psychiatric Clinics Basel (UPK), University of Basel, Switzerland*

[4]*Department of Psychology, University of Montana, Montana 59812, United States*

*Corresponding author:
Ulrike U. Bentele (ulrike.bentele@uni-konstanz.de)

ORCIDs:

Ulrike U. Bentele	https://orcid.org/0000-0002-3121-1957
Maria Meier	https://orcid.org/0000-0002-1655-5479
Jens C. Pruessner	https://orcid.org/0000-0002-8582-2980
Eva Unternaehrer	https://orcid.org/0000-0002-3507-1883

S1 Data preprocessing

Prior to the main analyses, psychological (subjective stress, negative affect, positive affect) and neuroendocrine data (cortisol) were preprocessed as follows: First, *missing values* were replaced (psychological data: 0%, cortisol: 2.43%). For cortisol, single missing values at the first timepoint were replaced by the sample's mean at that timepoint; missing values occurring at any timepoint during the experiment where imputed linearily by the mean of the individual's values prior and following that timepoint. There was no single missing value at the last timepoint. Two consecutive missing values in the period prior to the active part of the TSST (first to third timepoint) were replaced by the sample's mean at the respective timepoints; consecutive missing data following the TSST (fifth and sixth timepoint) were imputed linearily. Second, values that exceeded the sample's mean plus or minus 3 SD at a respective timepoint (limit values) were considered *outliers* and corrected to the maximum or minimum limit value to reduce their statistical impact (winsorizing; psychological data: 0% to 2.48%, cortisol: 2.64%). Third, in case of skewed data *transformations* were applied to meet the assumptions of parametric statistical models. Skewed cortisol levels were transformed applying the natural logarithm. Skewness in psychological data (subjective stress, negative affect) could not be successfully treated by transformation; thus, analyses were conducted with untransformed data and complemented by robust analyses. Finally, we calculated the area under the curve with respect to increase (AUCi, Pruessner et al., 2003), a common index of overall stress reactivity. Due to the different temporal dynamics of cortisol and subjective stress responses (Schlotz, 2008), the calculation of the AUCi was based on different time intervals: For cortisol, the $AUCi_{cort}$ includes the time interval from stressor onset (+0min) until end of recovery (+40min); for subjective stress, the $AUCi_{stress}$ includes the overall duration of the testing (-20 to +40min); for affect, the $AUCi_{NA}$ (negative affect) resp. $AUCi_{PA}$ (positive affect) are based on the time prior to the manipulation (-20min) to stressor cessation (+10min). Skewness in $AUCi_{cort}$ was adjusted by a natural log transformation of ($AUCi_{cort}$ + 340) (with +340 to have positive values only); as $AUCi_{stress}$ and $AUCi_{NA}$ could not successfully be transformed, untransformed data were used and complemented by robust analyses.

Table S2. Comparison of growth curve models for cortisol levels following step-wise incorporation of effects using Analyses of Variance.

number	model	model specification		*df*	*logLikelihood*	test	*df*	*Chi*^{2}	*p*
		simpler model	added effect						
1	FI			2	-477.59				
2	**RI**	FI	RI	3	-303.65	1 vs 2	1	347.90	<.001
3	**Fixed.time**	RI	Time	4	-295.43	2 vs 3	1	16.43	<.001
4	**Fixed.time^2**	RI	Time^2	5	-287.30	3 vs 4	1	16.27	<.001
5	Fixed.time^3	RI	Time^3	6	-286.82	4 vs 5	1	0.96	.328
6	**Random.time^1**	Fixed.time^2	random Time	7	-263.53	4 vs 6	2	47.54	<.001
7	**Random.time^2**	Fixed.time^2	random Time^2	13	-238.56	6 vs 7	6	49.94	<.001
8	AR	Random.time^2	covariance structure	14	-237.32	7 vs 8	1	2.47	.116
9	ER	Random.time^2	regulation	14	-237.80	7 vs 9	1	1.51	.219
10	ER x time^2	ER	regulation x Time^2	16	-235.83	9 vs 10	2	3.95	.139
11	MC	ER x time^2	MC	17	-235.70	10 vs 11	1	0.25	.620
12	MC x time^2	MC	MC x Time^2	19	-235.52	11 vs 12	2	0.37	.830
13	ER x MC	MC x time^2	regulation x MC	20	-235.25	12 vs 13	1	0.54	.463
14	ER x MC x time^2	ER x MC	regulation x MC x Time^2	22	-233.54	13 vs 14	2	3.41	.182
15	HS	ER x MC x time^2	hormonal status	25	-232.57	14 vs 15	3	1.94	.585

number	model	model specification		*df*	*logLikelihood*	test	*df*	Chi^2	*p*
		simpler model	added effect						
16	**HS x time²**	HS	hormonal status x Time²	31	-225.91	15 vs 16	6	13.32	.038

Note. Final model to evaluate (1) the success of the stress induction (manipulation check) was model 7, and (2) the interaction of MC and regulation (hypothesis) was model 16. Cortisol levels were transformed using the natural logarithm and baseline-adjusted. Significant improvement in model fit when models of increasing complexity were compared pairwise are printed in bold. *Time* represents the linear, $Time^2$ the quadratic, and $Time^3$ the cubic effect of time. Interactions are indicated by 'x'. FI = Fixed intercept, RI = random intercept, RS = random slope, AR = covariance structure, MC = maternal care, HS = hormonal status, ER = Emotion regulation. $N_{\text{subjects}} = 91$, $N_{\text{observations}} = 455$.

Table S3. Parameters of final growth curve models predicting changes in cortisol levels by time (basic model), MC, regulation condition and hormonal status (interaction model)

	basic model			interaction model		
Fixed effects	Estimate (*SE*)	*t*	*p*	Estimate (*SE*)	*t*	*p*
Baseline	0.16(0.06)	2.48	**.014**	0.20(0.19)	1.07	.288
Time	1.45(0.49)	2.96	**.003**	3.36(1.38)	2.44	**.015**
$Time^2$	-1.41(0.37)	-3.80	**<.001**	-0.77(1.08)	-0.71	.478
MC				-0.25(0.19)	-1.28	.202
Time x MC				-2.72(1.41)	-1.93	.054
$Time^2$ x MC				1.29(1.11)	1.16	.246
regulation				-0.31(0.22)	-1.39	.169
Time x regulation				-3.90(1.61)	-2.42	**.016**
$Time^2$ x regulation				0.68(1.26)	0.54	.590
Hormonal $status_{OC-FP}$				0.18(0.18)	1.00	.320
Hormonal $status_{OC-LP}$				0.20(0.17)	1.18	.240
Hormonal $status_{OC-UP}$				0.37(0.19)	1.97	.052
Time x Hormonal $status_{OC-FP}$				2.68(1.33)	2.02	**.044**
$Time^2$ x Hormonal $status_{OC-FP}$				-1.36(1.04)	-1.30	.193
Time x Hormonal $status_{OC-LP}$				-1.00(1.22)	-0.82	.412
$Time^2$ x Hormonal $status_{OC-LP}$				-1.67(0.96)	-1.74	.082
Time x Hormonal $status_{OC-UP}$				0.97(1.38)	0.70	.483
$Time^2$ x Hormonal $status_{OC-UP}$				-2.11(1.08)	-1.95	.052
MC x regulation				0.33(0.27)	1.22	.227
Time x MC x regulation				4.09(1.99)	2.06	**.040**
$Time^2$ x MC x regulation				-2.02(1.56)	-1.29	.197
Random effects	*SD*	correlation		*SD*	correlation	
Variance baseline	0.47	-		0.46	-	
Variance slope (Time)	3.47	-0.16		3.14	-0.20	
Variance slope ($Time^2$)	2.78	-0.66		2.61	-0.62	
Residuals	0.23	-		0.23	-	

Note. MC (levels: 0 = low MC, 1 = high MC), regulation condition (level: 0 = control, 1 = reappraisal) and hormonal status (levels: 0 = OC, 1 = follicular phase, 2= luteal phase, 3 = unclear phase) were entered as dummy coded factors. *Time* represents the linear, $Time^2$ the quadratic, and $Time^3$ the cubic effect of time. Interactions are indicated by ‘x’. Cortisol levels were transformed using the natural logarithm and baseline-adjusted. $N_{subjects}$ = 91, $N_{observations}$ = 455. MC = maternal care, *SE* = standard error, *SD* = standard deviation, OC = oral contraceptive intake, FP = follicular phase, LP = luteal phase, UP = unclear phase.

Table S4. Comparison of growth curve models for subjective stress levels following step-wise incorporation of effects using Analyses of Variance.

number	model name	model specification		*df*	*logLikelihood*	Test	*df*	*Chi*^{2}	*p*
		simpler model	added effect						
1	FI			2	-2738.50				
2	**RI**	FI	RI	3	-2730.58	1 vs 2	1	15.83	**<.001**
3	**Fixed.time**	RI	Time	4	-2705.20	2 vs 3	1	50.78	**<.001**
4	**Fixed.time^2**	RI	Time^2	5	-2611.60	3 vs 4	1	187.19	**<.001**
5	**Fixed.time^3**	RI	Time^3	6	-2564.76	4 vs 5	1	93.70	**<.001**
6	Random.time^1	Fixed.time^3	Random Time	8	-2564.54	5 vs 6	2	0.43	.808
7	**Random.time^2**	Fixed.time^3	Random Time^2	14	-2544.01	6 vs 7	6	41.07	<.001
8	**Random.time^3**	Fixed.time^3	Random Time^3	24	-2534.71	7 vs 8	10	18.60	.046
9	AR	Random.time^3	covariance structure	25	-2534.44	8 vs 9	1	0.54	.462
10	ER	Random.time^3	regulation	25	-2533.82	8 vs 10	1	1.76	.184
11	ER x time^3	ER	regulation x Time^3	28	-2532.81	10 vs 11	3	2.03	.567
12	MC	ER x time^3	MC	29	-2532.73	11 vs 12	1	0.16	.693
13	MC x time^3	MC	MC x Time^3	32	-2532.63	12 vs 13	3	0.21	.977
14	ER x MC	MC x time^3	regulation x MC	33	-2532.53	13 vs 14	1	0.21	.647
15	ER x MC x time^3	ER x MC	regulation x MC x Time^3	36	-2532.24	14 vs 15	3	0.56	.905

Note. Final model to evaluate the success of the stress induction to increase subjective stress levels (manipulation check) was model 8. Subjective stress levels were baseline-adjusted. Significant improvement in model fit when models of increasing complexity were compared pairwise are printed in bold. *Time* represents the linear, $Time^2$ the quadratic, and $Time^3$ the cubic effect of time. Interactions are indicated by ‘x’. FI = Fixed intercept, RI = random intercept, RS = random slope, AR = covariance structure, MC = maternal care, ER = Emotion regulation. $N_{subjects} = 93$, $N_{observations} = 651$.

Table S5. Parameters of final growth curve models predicting changes in subjective stress levels by time (basic model), MC and regulation condition (interaction model)

	basic model			interaction model		
Fixed effects	Estimate (*SE*)	*t*	*p*	Estimate (*SE*)	*t*	*p*
Baseline	7.98(0.81)	9.82	**<.001**	8.13(1.91)	4.26	**<.001**
Time	-107.19(12.07)	-8.88	**<.001**	-100.56(28.23)	-3.56	**<.001**
Time^2	-185.40(13.70)	-13.53	**<.001**	-188.23(32.23)	-5.84	**<.001**
Time^3	115.45(12.08)	9.56	**<.001**	166.26(28.34)	4.10	**<.001**
MC				0.78(2.37)	0.32	.744
Time x MC				14.66(35.13)	0.42	.677
Time^2 x MC				6.00(40.12)	0.15	.881
Time^3 x MC				-13.24(35.27)	-0.38	.707
regulation				-1.30(2.74)	-0.47	.637
Time x regulation				-16.95(40.54)	-0.42	.676
Time^2 x regulation				17.78(46.29)	0.38	.701
Time^3 x regulation				7.54(40.70)	0.19	.853
MC x regulation				-0.08(3.41)	-0.028	.982
Time x MC x regulation				-25.29(50.48)	-0.50	.617
Time^2 x MC x regulation				-30.73(57.63)	-0.53	.594
Time^3 x MC x regulation				13.12(50.68)	0.26	.800
Random effects	*SD*	correlation		*SD*	correlation	
Variance baseline	6.81	-		6.76	-	
Variance slope (Time)	62.76	-0.27		60.59	-0.30	
Variance slope (Time^2)	88.40	-0.91		87.66	-0.92	
Variance slope (Time^3)	62.87	0.54		61.46	0.57	
Residuals	10.12	-		10.13	-	

Note. MC (levels: 0 = low MC, 1 = high MC) and regulation condition (level: 0 = control, 1 = reappraisal) were entered as dummy coded factors. *Time* represents the linear, *Time^2* the quadratic, and *Time^3* the cubic effect of time. Interactions are indicated by ‘x’. Subjective stress levels were baseline-adjusted. $N_{\text{subjects}} = 93$, $N_{\text{observations}} = 651$. MC = maternal care, *SE* = standard error, *SD* = standard deviation.

Table S6. Parameters of final growth curve models in the low MC and high MC group predicting changes in cortisol levels by regulation condition and hormonal status

	Low MC model			High MC model		
Fixed effects	Estimate (*SE*)	*t*	*p*	Estimate (*SE*)	*t*	*p*
Baseline	0.21(0.24)	0.87	.387	-0.03(0.17)	-0.18	.860
Time	0.69(0.87)	0.80	.427	1.31(1.06)	1.24	.218
$Time^2$	-0.01(0.67)	-0.02	.983	0.07(0.87)	0.08	.936
regulation	-0.36(0.23)	-1.56	.132	0.02(0.16)	0.09	.927
Time x regulation	-2.09(0.82)	-2.54	**.013**	0.18(1.00)	0.18	.860
$Time^2$ x regulation	0.25(0.64)	0.39	.694	-0.91(0.82)	-1.11	.268
Hormonal $status_{OC-FP}$	0.22(0.31)	0.72	.476	0.15(0.23)	0.68	.499
Hormonal $status_{OC-LP}$	0.11(0.28)	0.41	.688	0.25(0.21)	1.18	.242
Hormonal $status_{OC-UP}$	0.59(0.34)	1.72	.097	0.28(0.23)	1.24	.219
Time x Hormonal $status_{OC-FP}$	2.44(1.12)	2.18	**.031**	1.60(1.37)	1.16	.245
$Time^2$ x Hormonal $status_{OC-FP}$	-1.84(0.86)	-2.13	**.036**	-0.32(1.12)	-0.29	.775
Time x Hormonal $status_{OC-LP}$	1.78(1.02)	1.75	.083	-2.65(1.27)	-2.09	.038
$Time^2$ x Hormonal $status_{OC-LP}$	-1.37(0.79)	-1.73	.086	-1.09(1.04)	-1.05	.296
Time x Hormonal $status_{OC-UP}$	1.89(1.25)	1.52	.131	-0.144(1.38)	-0.10	.917
$Time^2$ x Hormonal $status_{OC-UP}$	-1.21(0.96)	-1.25	.214	-1.65(1.13)	-1.46	.146
Random effects	*SD*	correlation		*SD*	correlation	
Variance baseline	0.57	-		0.45	-	
Variance slope (Time)	1.70	0.11		2.58	-0.29	
Variance slope ($Time^2$)	1.06	-0.63		2.35	-0.62	
Residuals	0.22	-		0.23	-	

Note. Regulation condition (level: 0 = control, 1 = reappraisal) and hormonal status (levels: 0 = OC, 1 = follicular phase, 2= luteal phase, 3 = unclear phase) were entered as dummy coded factors. *Time* represents the linear and $Time^2$ the quadratic effect of time. Interactions are indicated by 'x'. Cortisol levels were transformed using the natural logarithm and baseline-adjusted. Models included $N_{subjects}$ = 31 with $N_{observations}$ = 155 (low MC group) respective $N_{subjects}$ = 60 with $N_{observations}$ = 300 (high MC group). *SE* = standard error, *SD* = standard deviation, OC = oral contraceptive intake, FP = follicular phase, LP = luteal phase, UP = unclear phase.

Table S7. Comparison of growth curve models for negative affect levels following step-wise incorporation of effects using Analyses of Variance.

number	model	model specification		*df*	*logLikelihood*	test	*df*	*Chi*^{2}	*p*
		simpler model.	added effect						
1	FI			2	-827.09				
2	**RI**	FI	RI	3	-815.20	1 vs 2	1	23.78	**<.001**
3	**Fixed.time**	RI	Time	4	-789.17	2 vs 3	1	52.07	**<.001**
4	Fixed.time^2	RI	Time^2	5	-789.12	3 vs 4	1	0.09	.770
5	**Random.time[1]**	Fixed.time	random Time	6	-753.24	3 vs 5	2	71.85	**<.001**
6	CAR	Random.time[1]	covariance structure	7	-753.25	5 vs 6	1	0.02	.901
7	ER	Random.time[1]	regulation	7	-753.24	5 vs 7	1	0.01	.914
8	ER x time[1]	ER	regulation x Time[1]	8	-752.84	7 vs 8	1	0.79	.374
9	MC	ER x time[1]	MC	9	-752.77	8 vs 9	1	0.14	.701
10	MC x time[1]	MC	MC x Time[1]	10	-751.79	9 vs 10	1	1.96	.162
11	ER x MC	MC x time[1]	regulation x MC	11	-751.77	10 vs 11	1	0.06	.811
12	ER x MC x time[1]	ER x MC	regulation x MC x Time[1]	12	-751.40	11 vs 12	1	0.73	.393

Note. Final model to evaluate changes in negative affect was model 5. Negative affect levels were baseline-adjusted. Significant improvement in model fit when models of increasing complexity were compared pairwise are printed in bold. *Time* represents the linear, *Time*^{2} the quadratic effect of time. Interactions are indicated by 'x'. FI = Fixed intercept, RI = random intercept, RS = random slope, CAR = covariance structure, MC = maternal care. ER = Emotion regulation. $N_{subjects} = 93$, $N_{observations} = 279$.

Table S8. Comparison of growth curve models for positive affect levels following step-wise incorporation of effects using Analyses of Variance.

number	model	model specification		*df*	*logLikelihood*	test	*df*	*Chi*^{2}	*p*
		simpler model.	added effect						
1	FI			2	-889.89				
2	**RI**	FI	RI	3	-853.73	1 vs 2	1	72.32	**<.001**
3	**Fixed.time**	RI	Time	4	-847.32	2 vs 3	1	12.83	**<.001**
4	Fixed.time^2	RI	Time^2	5	-847.26	3 vs 4	1	0.12	.728
5	**Random.time[1]**	Fixed.time	random Time	6	-835.02	3 vs 5	2	24.59	**<.001**
6	CAR	Random.time[1]	covariance structure	7	-835.03	5 vs 6	1	0.01	.937
7	ER	Random.time[1]	regulation	7	-834.86	5 vs 7	1	0.33	.563
8	ER x time[1]	ER	regulation x Time[1]	8	-834.70	7 vs 8	1	0.31	.577
9	MC	ER x time[1]	MC	9	-834.61	8 vs 9	1	0.18	.669
10	MC x time[1]	MC	MC x Time[1]	10	-834.17	9 vs 10	1	0.87	.351
11	ER x MC	MC x time[1]	regulation x MC	11	-834.15	10 vs 11	1	0.04	.843
12	ER x MC x time[1]	ER x MC	regulation x MC x Time[1]	12	-833.24	11 vs 12	1	1.82	.177

Note. Final model to evaluate changes in positive affect was model 5. Positive affect levels were baseline-adjusted. Significant improvement in model fit when models of increasing complexity were compared pairwise are printed in bold. *Time* represents the linear, $Time^2$ the quadratic effect of time. Interactions are indicated by 'x'. FI = Fixed intercept, RI = random intercept, RS = random slope, CAR = covariance structure, MC = maternal care, ER = Emotion regulation. N_{subjects} = 93, $N_{\text{observations}}$ = 279.

Table S9. Parameters of final growth curve models predicting negative and positive affect by time

	Negative affect model			Positive affect model		
Fixed effects	Estimate (*SE*)	*t*	*p*	Estimate (*SE*)	*t*	*p*
Baseline	0.06(0.30)	0.18	.856	-0.08(0.54)	-0.14	.889
Time	0.13(0.02)	6.17	**<.001**	0.07(0.02)	3.01	**.003**
Random effects	*SD*	correlation		*SD*	correlation	
Variance baseline	2.21	-		4.55	-	
Variance slope (Time)	0.17	0.03		0.17	-0.25	
Residuals	2.24	-		2.89	-	

Note. Time represents the linear effect of time. Negative and positive affect levels are baseline-adjusted. N_{subjects} = 93, $N_{\text{observations}}$ = 279. *SE* = standard error, *SD* = standard deviation.

Table S10. Summary of results concerning the effects of MC, regulation and the interaction on subjective-emotional reactivity

	parametric[b]		non-parametric[c]	
	statistics	*p*	statistics	*p*
Subjective stress reactivity[a]				
regulation	F(1,89) =0.31	.578	0.09	.765
MC	F(1,89)=0.13	.721	0.005	.947
MC x regulation	F(1,89)=0.002	.964	0.01	.927
Negative affect reactivity[a]				
regulation	F(1,89)=0.84	.363	0.57	.456
MC	F(1,89)=2.12	.149	1.59	.219
MC x regulation	F(1,89)=0.79	.375	0.92	.348
Positive affect reactivity[a]			-	-
regulation	F(1,89)=0.96	.329	-	-
MC	F(1,89)=0.90	.344	-	-
MC x regulation	F(1,89)=0.28	.596	-	-

Note. Inferential statistics for the effect of MC, regulation and the MC by regulation interaction on stress reactivity. Effect size represents η_p^2. Interactions are indicated by ‘x’.

[a] operationalized by the Area under the curve with respect to increase for subjective stress (AUC_{stress}, between -20 to +40min) and negative and positive affect (AUC_{NA} resp. AUC_{PA},between -20 to +10min).

[b] 2(MC) x 2(regulation) ANOVA.

[c] robust 2(MC) x 2(regulation) ANOVA based on 20% trimmed means.

Table 11. Comparison of growth curve models for cortisol levels following step-wise incorporation of effects using Analyses of Variance.

number	model	model specification		*df*	*logLikelihood*	test	*df*	*Chi*^{2}	*p*
		simpler model	added effect						
8	Random.time^2			13	-238.57				
9	ER	Random.time^2	regulation	14	-237.80	8 vs 9	1	1.51	.219
10	ER x time^2	ER	regulation x Time^2	16	-235.83	9 vs 10	2	3.95	.139
11	MC	ER x time^2	MC	17	-235.58	10 vs 11	1	0.49	.485
12	MC x time^2	MC	MC x Time^2	19	-235.31	11 vs 12	2	0.55	.761
13	ER x MC	MC x time^2	regulation x MC	20	-235.30	12 vs 13	1	0.01	.903
14	ER x MC x time^2	ER x MC	regulation x MC x Time^2	22	-232.86	13 vs 14	2	4.88	.087
15	HS	ER x MC x time^2	hormonal status	25	-232.05	14 vs 15	3	1.62	.655
16	HS x time^2	HS	hormonal status x Time^2	31	-225.77	15 vs 16	6	12.55	.051

Note. Stepwise modeling of cortisol trajectories starting from the basic model (random.time^2) to evaluate the interaction of *MC* and *regulation* (hypothesis). Cortisol levels were transformed using the natural logarithm and baseline-adjusted. *MC* was added as continuous, *regulation* as binary predictor. *Time* represents the linear, *Time*^{2} the quadratic, and *Time*^{3} the cubic effect of time. Interactions are indicated by 'x'. FI = Fixed intercept, RI = random intercept, RS = random slope, AR = covariance structure, MC = maternal care, HS = hormonal status, ER = Emotion regulation. $N_{subjects} = 91$, $N_{observations} = 455$
